# Comment on “Topically Applied Connective Tissue Growth Factor/CCN2 Improves Diabetic Preclinical Cutaneous Wound Healing: Potential Role for CTGF in Human Diabetic Foot Ulcer Healing”

**DOI:** 10.1155/2015/512959

**Published:** 2015-09-20

**Authors:** Hongling Li, Cong Cao, Ai Huang, Yi Man

**Affiliations:** ^1^State Key Laboratory of Oral Diseases, Sichuan University, Chengdu 610041, China; ^2^Department of Stomatology, China-Japan Friendship Hospital, No. 2 Yinghuayuan East Road, Beijing 100029, China; ^3^Department of Oral Implantology, West China Hospital of Stomatology, Sichuan University, Chengdu 610041, China

## Abstract

A recent paper in this journal, presented a novel method by topical application of growth factors in stimulating diabetic cutaneous wound healing that caught our attention. We believe that the experimental method in the article is efficient and creative, but it also has some controversies and shortcomings to be discussed. We noted that the authors used “Tegaderm” as a semiocclusive dressing film and stated that it exerted a “splinting effect” on the wound margins and controlled contraction. Indeed, the “Tegaderm” itself can serve as a dressing film to isolate the wound bed with outside environments while the “splinting effect” is mainly achieved by adding silicone splints around the wound. Considering the unique properties of silicone splints and “Tegaderm,” our experimental group propose an alternative method named “combined-suturing” technique that is not only suturing the silicone splints but also securing the “Tegaderm” around the wound. The specific reasons and operative procedures are explained in detail in this letter.

In a recent issue of this journal, we read the article by Henshaw et al. with great interest, focusing on an efficient method of the topical application of Connective Tissue Growth Factor (CTGF)/CCN2 in accelerating cutaneous wound healing in a diabetic rat model [1].

We would like to comment on a concept presented by the authors in the Discussion part that the semipermeable film dressing “Tegaderm” exerts a “splinting effect” on the wound margins and contraction, thus promoting healing through reepithelialization, which better approximates human wound healing, rather than through contraction which predominates in nonsplinted rodent wounds (page 6, 2nd paragraph) [1]. For the murine excisional wounds, a widely utilized model in wound healing studies still does have some disadvantages that cannot totally parallel human wound healing [2]. In the early stage of healing, the mechanism of wound closure is mainly by reepithelialization in human while it is by skin contracture in rodents [3]. To imitate human skin wound healing, donut-shaped silicone splints have always been applied to present a “splinting effect” on the wound margins and control muscle contractions [2, 4–7]. Consequently, from our perspective, it is the silicone splints rather than “Tegaderm” that provide the “splinting effect” for the murine dorsal cutaneous wounds. What is more, we noticed that the authors had put forward the conclusion from the study of Peplow et al. [8]. However, the review only mentioned that the use of “Tegaderm” can keep the wound moist and facilitate reepithelization, thus creating conditions similar to human skin healing, but did not support the concept of “splinting effect” mentioned above.

Meanwhile, we noted that “Tegaderm” was used to cover each ulcer and secured with Hypafix tape peripherally in the article. Both of the two materials are soft and thin with limited adhesive strength. What is more, as a kind of energetic animal, rat would like to bite or scratch the foreign materials on the dorsum after revival. Therefore, the “Tegaderm” and Hypafix were inclined to fall from the rat dorsum easily. The researchers had to replace the “Tegaderm” and Hypafix at every interval of 24 h (the methods as described in the article). As a result, an excellent fixation is quite difficult to achieve by joint application of “Tegaderm” and Hypafix, let alone a “splinting effect” without any other treatments.

It is worth mentioning that our experimental group is making similar efforts in utilizing the rat dorsal wounds model to study the effects of growth factors on promoting wound healing. As described above, rats possess a panniculus carnosus and loose skin layer which present a significant contractile capacity [2]. In order to minimize the contraction of the rat dorsal muscle by making the wounds healing mainly by reepithelialization, a donut-shaped silicone splint is being placed around each dorsal wound. Additionally, the semiocclusive transparent film “Tegaderm” is being used to provide a moist wound environment for stimulating healing much faster than in dry conditions and also act as a promoter in wound closure and collagen deposition and reduces the scar formation [9]. We apply both of silicone splints and “Tegaderm” on the rat dorsal wounds to obtain better and faster healing mainly through reepithelialization. In previous studies, the donut-shaped silicone splints around the wound would be fixed by bonding or suturing, while “Tegaderm” covered on the wound would be mainly fixed by bonding. But, from our prospective, the bonding force is pretty limited, so silicone splints and “Tegaderm” will be easy to come off and then out of their action. After selecting and modifying the old methods, we presented a new one which is named “combined-suturing” technique that is not only suturing the silicone splints but also securing the “Tegaderm” tightly around the rat dorsal wound. We wish this “combined-suturing” technique could provide a better fixation than single sutured silicone splints with bonded “Tegaderm” method, let alone “dual-bonding” method. Therefore, the innovation point of our method is the “combined-suturing” fixation pattern, for achieving a better fixation and a longer maintenance of these two materials on the rat dorsum.

We would like to describe our procedures in detail, hoping that some innovations will help the investigators to choose appropriate methods for the experiments relevant to the widely used excisional murine wound model. The main procedures include the following: (1) predispose the shaped rubber stamp (diameter of 8 mm) and donut-shaped silicone splints (inner diameter of 10 mm, outer diameter of 12 mm, fabricated from 1 mm thick silicone sheet) by soaking in 75% alcohol solution for 1 hour; (2) pull out the silicone splints from the disinfectant and let them dry out in the air, then glue them to the “Tegaderm” inner surface; (3) clip the silicone splint with the “Tegaderm” layer together (Figure 1(b)); (4) position the rat in ventral recumbency after anesthesia, shaving preparation, and sterilization (Figure 1(c)); (5) measure and locate the wounds with the shaped rubber stamp inked with gentian violet (Figure 1(d)) and then create four full-thickness wounds including the panniculus carnosus layer bilaterally on each rat dorsum (Figures 1(e) and 1(f)); (6) secure the two materials together with 3-0 nylon sutures for 6 sutures which interrupted the “Tegaderm” and surrounded the silicone splint around every wound (Figures 1(g) and 1(h)). With such a well-defined, controlled, and splinted full-thickness wound model, we could investigate the efficacy of growth factors or other treatments in stimulating wound closure mainly by reepithelialization. And, undoubtedly, the simultaneous sutured “Tegaderm” could minimize multiple confounding factors leading to its inconsistent maintenance efficiently.

Finally, we acknowledge Henshaw et al. for their indelible work in the field of topical application of growth factors promoting wound healing, especially for chronic wounds in diabetic models, which may be of far-reaching significance for the future clinic therapies.

## Figures and Tables

**Figure 1:**
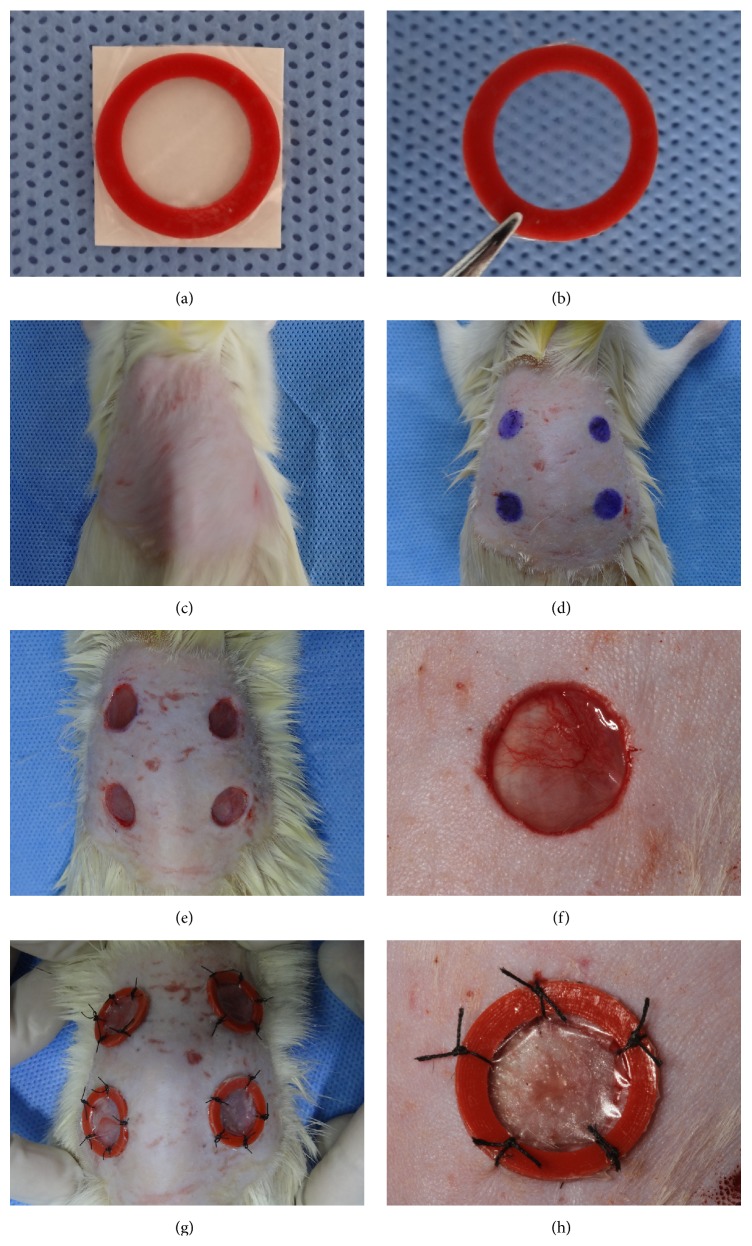
Procedures of creating wounds and securing silicone splints and “Tegaderm”: (a) glue the silicone splint to the “Tegaderm” inner surface; (b) clip the silicone splint with the “Tegaderm” layer together; (c) treat the rat with conventional anesthetization, skin preparation and sterilization, then put it in ventral recumbency; (d) measure and locate the wounds with the shaped rubber stamp inked with gentian violet; (e) create four full-thickness wounds on the rat dorsum; (f) gross appearance of single wound; (g) suture the silicone splint with “Tegaderm” together; (h) gross appearance of single wound covered with silicone splint and “Tegaderm” sutured together.
